# Congruity of Virtual Reality In-Game Advertising

**DOI:** 10.3389/fspor.2021.728749

**Published:** 2021-10-12

**Authors:** Joshua M. Lupinek, Jinhee Yoo, Eugene A. Ohu, Eric Bownlee

**Affiliations:** ^1^Feliciano School of Business, Montclair State University, Montclair, NJ, United States; ^2^Dahlkemper School of Business, Gannon University, Erie, PA, United States; ^3^Virtual Human Computer Interaction Lab, Lagos Business School, Pan-Atlantic University, Lagos, Nigeria

**Keywords:** virtual reality (VR), In-Game Advertising (IGA), marketing, video gaming, congruity, telepresence

## Abstract

With virtual reality (VR) video game users beginning to see beta advertisements within game play, this conceptual article adds a needed digital and interactive marketing research foundation to the new construct of VR in-game advertising (IGA) activation. New consumer VR technology continues to disrupt traditional media as a $7.7 billion USD industry, that is expected to reach $57.55 billion by 2027. As such, marketing researchers must continue to evolve and understand the interdisciplinary VR research evolution as many VR users are likely to view IGA as intrusive. IGA and VR are not new constructs, but IGA within VR is unique as VR consumers have not yet experienced VR IGA intrusion. This article utilizes a sport marketing focus to provide an industry specific set of examples for the reader, however this article can be applied to broader fields including communications and interactive marketing. The main contributions of this article are 2-fold. First the development of a *VR In-Game Advertising Congruity Framework* is developed through a review of the literature and application to VR IGA in the topical areas of *congruity* of the IGA, *interactivity* of the IGA, *intrusiveness* of the IGA, *realism* of the experience, *telepresence, brand awareness*, and *attitude* toward the IGA. Secondly, a proper VR context definition of telepresence is provided through review of the literature that takes into account the interaction of a VR participant. This article aims to aid marketers in making informed IGA development decisions through strategic choice, via a centralized VR IGA congruity framework, that not only enhances brand awareness, but leaves participants with a favorable attitude toward the IGA to increase sales activation.

## Introduction

The purpose of this conceptual article is to add a much needed timely digital and interactive marketing research foundation to virtual reality (VR) in-game advertising (IGA). In July 2021 people with Oculus Quest headsets are scheduled to start seeing beta advertisements in virtual reality (Olson, 2021). IGA is not a new construct, but IGA in VR is as unique as this conceptual analysis since VR consumers have not yet experienced VR IGA intrusion. As this new consumer VR technology continues to disrupt traditional media, marketing researchers must continue to evolve and understand the interdisciplinary VR research evolution. This work uses a sport video game context to examine the emerging VR gaming industry, the user intrusion anticipated by IGA, and the VR IGA's sport marketing ramifications across disciplines such as advertising.

**Figure 1:**
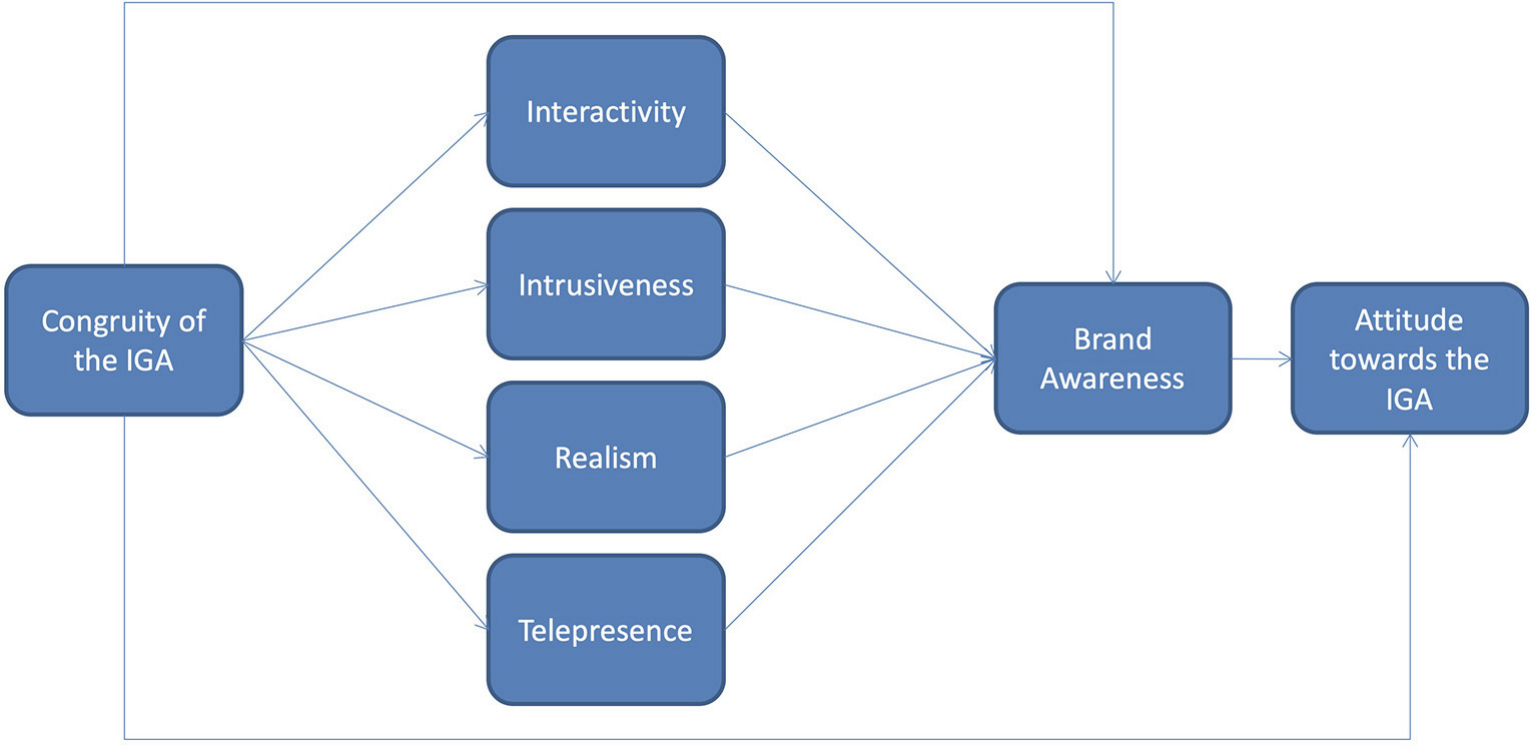
VR in-game advertising congruity framework.

Due to the novelty of VR as well as the importation of ideas from fields other than sport marketing, there are numerous conceptual and definitional inconsistencies in the literature around terms such as presence, spatial presence, and telepresence along with elements related to the immersive characteristics of VR technology. This concept is practically relevant to the design and evaluation of media products and human-computer interactions, such as VR games and IGA. For this reason, scholars have paid much attention to presence, spatial presence, and telepresence and suggested various definitions since the mid-90s (Lee, 2004). One major goal of this work is an attempt to provide some clarity of common IGA terms within the VR context as consumer experience VR IGA for the first time in 2021 in order to enhance successful communication among scholars. Topical areas of *congruity* of the IGA, *interactivity* of the IGA, *intrusiveness* of the IGA, *realism* of the experience, *telepresence, brand awareness*, and *attitude* toward the IGA are unraveled within the literature. *Congruity* was identified as the centralized variable of the VR IGA congruity framework based on the topical VR context with IGA referring to the compatibility, agreement, or harmony between the elements of the game and of the VR advertisement. Two main contributions of this article are the development of a *VR In-Game Advertising Congruity Framework* (see Figure 1) and a proper *telepresence* definition through review of the literature with a VR active participant context.

It is important to note that augmented reality (AR), digital overlay on top of a real-life first person view, often goes hand in hand with VR discussions (i.e., AR/VR) and that this article focuses solely on VR technology and research opportunities for VR marketing exploration. VR uses computing technology but its technical properties far exceed those of a computer, giving it potentially superior applications in many fields. As a medium of work and entertainment, VR (like the television and the computer, powered by the Internet) has great potential as a sport marketing medium that brings advertisers in contact with target “eye balls,” only VR is more interactive and more immersive.

Just like advertisers have targeted sport video games as an outlet for showcasing their products and services, VR holds even more promises, only if advertisers can get assurance that they are properly and adequately engaging their target audience. Brand awareness is therefore a key goal that advertisers want to achieve in product placement (Karrh et al., 2003), which in the case of VR is through IGA. In addition to providing greater clarity to the concept of IGA within VR, this study leverages on the state achieved by the participant to analyze the VR user's *attitude* toward the IGA with a view toward proposing and contributing effective evaluation of interactive marketing initiatives within VR game environments. This improved knowledge of the participant experience, within the VR gaming environment, aims to assist sport marketers in making informed IGA development decisions through strategic choice that not only enhances brand awareness, but leaves participants with a favorable attitude toward the IGA to increase sales activation.

## Literature Review

In this study, a sport *VR In-Game Advertising Congruity Framework* is developed and proposed based on an extensive review of literature to guide researchers and marketers in the sport VR gaming industry (see Figure 1). Each variable of this conceptual framework is developed and supported within the literature review below for future empirical research lines and practical marketer application. This research team believes that insufficient attention has been dedicated to the relationship between sport video games along with sport consumer behavior and this is especially evident in the sport VR video game setting. This is despite the fact that the sport video game industry is a growing, multi-billion dollar industry with significant recent growth in the sport VR area (Hong and Magnusen, 2017). For instance, the most popular sport video game in the world, FIFA, generated over $1.6 billion in revenue for EA Sports in the 2020 and this accounts for over 25% of the total revenue for the company (Murphy, 2021). The majority of this revenue came from FIFA Ultimate Team and a FIFA VR game for PlayStation 5 that has been released after years of development. Consequently, a sport-related VR gaming environment is utilized to discuss the variables of *congruity* of the IGA, *interactivity, intrusiveness, realism, telepresence, brand awareness*, and *attitude* toward the IGA throughout the general business and advertising literature.

### Congruity of the IGA

Advertisers desire that notwithstanding the primary task the player is involved in, they may notice and remember the in-game Ad. Kim and Ko (2019) limited-capacity model of attention suggests that it may however be a challenge for players to focus equally on the game as well as on the IGA. Game players can be selectively focused (Kahneman, 1973) or more or less intensely focused (Olshavsky, 1994), and since they can be exhausted, cognitive resources would have to be freed from a primary task, in order for spare capacity to be available for a secondary task.

Congruity may influence the ease with which players are able to share attention in this way. Lee and Faber (2007) further suggest that the interplay between primary task capacity and spare capacity may further explain how product placement in games affect brand memory. Congruity in a digital media context refers to the harmony between the elements it contains. These elements include visual or verbal elements of an IGA (Heckler and Childers, 1992; an IGA and the in-game context (Moorman et al., 2007) or harmony between the Ad sponsor and the event being sponsored (De Pelsmacker et al., 2019).

The causes of the perceived congruity can be intrinsic to the game, a property of the IGA, or in the user. Congruity may also be an aspect of the game structure, and thus can be perceived as simulation, gameplay or game narrative (Verberckmoes et al., 2016). IGA congruity may also be *thematic*—harmony between the game elements and the IGA (Lee and Faber, 2007), such as displaying an IGA of a sport related brand inside a sport VR game; advertising an energy drink within a space game, where the player's need for virtual energy may be a cue to the physical body's need for energy from an energy drink.

There is research suggesting that congruent information is better remembered than incongruent ones (Lambert, 1980; Moorman et al., 2007), the argument being that when people encounter new information, they try to match it with pre-existing schema. If the two information sets match, the new one would be absorbed into the old schema and thus be more easily remembered.

Other authors argue the contrary: that incongruent information is more likely to be remembered, because not matching with pre-existing information or schema, they tend to stand out, making them more easily remembered (Heckler and Childers, 1992; Russell, 2002; Dimofte et al., 2011). Despite the differences, the mechanism of effect on the memory seem to be based on both the degree of congruity and the amount of cognitive processing resources available to the individual.

We suggest that the same mechanism may explain the effect of IGA within VR on brand memory and awareness. What is at issue here though is the effect that this attention dispersal might have on the player based on their perception of harmony in the game play experience, and hence perceptions of congruity within the VR environment. Positive gameplay experience such as in a low cognitive load, aesthetic pleasure derived from a good graphics design, the absence of vestibular disturbances like motion sickness, would contribute to a mental, affective, and physical perception of congruence by the player.

### Interactivity of the IGA

Interactivity is the level to which users can change the form and content of a mediated environment in real time (Steuer, 1992). Interactivity in the context of VR video games is how actively involved the player is in the activities in and outcome of the interaction. They are thus able to modify their in-game experience and change the course of the game. For instance, a player in a VR auto racing game may choose a car with specific attributes such as a higher top speed, but slower acceleration; choices that can change their playing experience (Herrewijn and Poels, 2014). IGA marketers can also take advantage of the possibilities and expectations of interactivity in VR to have players be more actively involved with their ads, which can be made a part of the game play experience (Nelson et al., 2004; Lee and Faber, 2007; Lee et al., 2014). For example, a player may be able to customize their in-game experience within a football game by picking a branded uniform for their team or a player in a NASCAR themed racing game, may be able to virtually drink a Monster Energy drink to enhance their in-game focus (Pelsmacker et al., 2019).

Previous research has demonstrated that this IGA brand interaction typically leads to positive outcomes for brands and game developers as users want to play games with interactive advertising more often and the user may exhibit a higher level of brand awareness than players exposed to passive, non-interactive IGA (Wu and Liu, 2007; Wu et al., 2008; Pelsmacker et al., 2019). Papadopoulos (2020) found that VR gamers had a higher level of recognition of both familiar and unfamiliar brands when they had brand interactions with a VR environment. In short, participant interactivity with the IGA has enhanced brand awareness as opposed to lower level of brand awareness for participants merely “viewing” or being “exposed” to the IGA.

Research has also shown interactivity to be a multidimensional construct. There is as yet little consensus in the literature regarding the dimensions of interactivity as it relates to IGA (Steuer, 1992; Gao et al., 2009). Liu and Shrum (2002) identified three dimensions of interactivity related to advertising including active control, two-way communication, and synchronicity. These three dimensions of interactivity have been included in multiple mobile and VR video game studies and Gao et al. (2009) added connectedness, playfulness, and interpersonal communication as additional dimensions of interactivity related to mobile video game IGA.

### Intrusiveness of the IGA

According to previous research, a non-congruent IGA is likely to be intrusive. An intrusive IGA will augment the experience of incongruence, which produces an outcome that increases the likelihood of the IGA being noticed, stored in memory and recalled. Intrusiveness is defined as “the degree to which advertisements in a media vehicle interrupt the flow of an editorial unit” (Ha, 1996, p. 77). Editorial units in the digital media context should be broadly defined to include all media types including VR sport games.

We define intrusiveness in the context of VR video games as the degree to which advertisements embedded in the game do not conform with the digital medium's reality and/or impact the participant's VR experience. For instance, a player in a VR soccer game may expect to see advertisements embedded around the arena matching the real-world arena. However, the player may not expect there to be additional advertisements in non-traditional places such as on the goal posts and may place virtual blame on the IGA for the intrusive experience. With the unrestricted nature of VR game environments providing an unparalleled amount of advertising inventory, advertisers have an opportunity to push the envelope while not incurring intrusiveness “that occurs when an audience's cognitive processes are interrupted” (Li et al., 2002, p. 39). As such, enhanced brand awareness and recall of an intrusive IGA may produce a negative attitude toward the IGA resulting in decreased purchase intentions and actual purchase.

Marketers must be cognizant of new VR advertising inventory, but be wary of the temporal, visual, and flow characteristics of advertisements that consumers find intrusive so that negative responses are minimized (Riedel et al., 2018). For example, it would be realistic in a VR basketball game to have the participant's players within the game experience fatigue due to time between whistles and hydration. This presents an opportunity for advertisers to activate potential sponsors such as Gatorade where the participant has a chance to enhance their player's strength during timeouts by giving them Gatorade instead of water. However, this brand activation comes with a price and may be considered intrusive if not properly embedded or if this enhanced stamina is only available if you watch a 10 s commercial. It is worth noting that Poels et al. (2013) found a positive relationship between game players and their attitude toward IGA intrusiveness based on IGA context authenticity. As such in this context, we thus observe a parallel between intrusiveness and a lack of congruity. That which is incongruous can be so because it is intrusive. Likewise, because it is intrusive, the end result would be incongruity.

### Realism of the Experience

IGAs, congruence in function, lifestyle, image or advertising, add to the realism of the game experience. Due to a natural harmony of components of the IGA and the game environment, the senses are made to believe that the VR experience is real, rather than simulated. Realism is the element of VR media that receives the most attention, but is comparatively limited in empirical research. With realism being the driving industry force behind VR game development and advertiser activation, perceived realism should be a key focus as it can influence mental processing of media messages, attitudes and behavior, in some cases intensifying effects (Potter, 1988).

Krcmar et al. (2011) found that video game realism correlates with attention retention outcomes. As such, integrated VR advertisements with improved graphics and enhanced graphical realism may be more salient to players and thus, lead to more identification, and a greater sense of being “in” the game experience (Tamborini, 2000). For example, a participant playing a VR basketball game in a crystal-clear VR environment, should see a realistic in-game advertising experience complete with current jersey sponsors and in-arena sponsor signs. More specifically, a gamer playing an NBA VR game with the Denver Nuggets team should see Western Union prominently placed on the player's jerseys as this is current sponsor of this team at time of writing. Gamers demand realism in their gaming experience and this sense of realism may have a significant impact on the brand awareness of sponsoring companies.

Authenticity has also been found to impact participants' perceptions of game play realism (Malliet, 2006). Hall (2003) stated that events or behaviors are defined as plausible when they have “the potential to occur in the real world” (p. 629). Tavinor (2019) found that participants' sense of being within the world, the realistic appearance of the world's environments, and the feeling of joy, anxiety, and fear provoked by the events depicted, all make for a greater impression in VR. As such, sport VR game advertisers must place importance on exposing participants to a higher degree of visual realism, as visual realism experience has been correlated to a stronger sensation of presence (Hvass et al., 2017).

### Telepresence

Telepresence is a term derived from presence and it refers to “the mediated perception of an environment” whereas presence is “the natural perception of an immediate physical environment” (Steuer, 1992, p. 6). That is, contrary to the experience of “being here,” telepresence is the experience of “being there” or being in parallel space through a specific computer-mediate vehicle (Faiola et al., 2013). This definition has guided a stream of studies as researchers have adopted telepresence within their work in the fields of VR, marketing communication, web uses, and consumer behavior (e.g., Klein, 2003; Nah et al., 2011; Hyun and O'Keefe, 2012; Kim and Ko, 2019).

A conceptual study of Lombard and Ditton (1997) on presence has guided several presence and telepresence research studies. They defined presence as “the perceptual illusion of non-mediation” as the same concept as telepresence. That is, when the individual feels a sense of presence, the individual does not perceive or acknowledge the existence of a medium in his/her communication environment and responds as he/she would if the medium were not there in a form of illusion of non-mediation. They conceptualized presence using six aspects, social richness, realism, transportation, immersion, social actor within medium, and medium as social actor. This multi-dimensional view on presence has provided a conceptual foundation for telepresence studies and now needs clarification for VR adoption.

When applying Lombard and Ditton's (1997) conceptualization of presence, researchers have defined telepresence with a focus on a different dimension (e.g., transportation, immersion, etc.) or used different subscales to reflect their contexts. Lombard and Ditton argued that among the aspects of presence, transportation has the longest history and has been “often used in discussions of VR, which takes users to virtual environment and leads to the suspension of disbelief that they are in a world other than where their real bodies are located” (Slater and Usoh, 1993, p. 222). For this reason, the transportation aspect has taken a central role when defining telepresence (e.g., Held and Durlach, 1992; Biocca and Levy, 1995; Steuer, 1995).

As various media technologies have advanced, so has the level of immersion. Therefore, several researchers have stressed *immersion* as a dimension of telepresence. Mollen and Wilson (2010) proposed that telepresence is characterized by cognitive and sensory arousal, control, and immersion. In their study, immersion referred to perceiving oneself to be steeped in and interacting with an environment that sustains a continuous stream of stimuli and experiences in their conceptual work. Furthermore, some researchers even have used telepresence and immersion interchangeably (e.g., McGloin et al., 2011, 2013; Mcgloin et al., 2015; Nelson et al., 2013), furthering the need for a proper VR-based definition of telepresence. In a game-based learning study, Faiola et al. (2013) highlighted the role of telepresence to enhance a user's sense of being totally immersed in a virtual space. They suggested that virtual world users often feel completely immersed in the interactivity of a game, losing their sense of time, while feeling a heightened sense of pleasure, or what has been considered the gamers' optimal experience.

Although the work of Steuer (1992), Kim and Biocca (1997), and Lombard and Ditton (1997) have primarily guided telepresence research, it is critical to revisit how to define telepresence in the head mounted display (HMD) VR gaming setting due to its fast technological advancement, growing market demand, and its applicability in various areas past VR sport games, such as training, entertainment, education, health treatment, etc. Previous studies have focused on different aspects of telepresence in line with Lombard and Ditton's (1997) work, but this inconsistency has caused substantial confusion to researchers. Consequently, we need to reconsider the definition of telepresence that can be particularly applied to the HMD VR context.

When defining telepresence in the application of HMD VR, we need to pay attention to the notion that “being there” as a spectator is not the same as “being there” as an actor (Kim and Biocca, 1997; Klein, 2003; Nelson et al., 2013). In the current upward trend of highly interactive media, VR gaming is one of the most relevant areas that require close interactions between technology and users to optimize gaming experiences. It is apparent that telepresence plays a significant role affecting sport gaming experiences, and telepresence deserves a great amount of attention from both researchers and practitioners to benefit consumers, the industries, and the overall body of business knowledge.

As such, the following clarifying VR definition has been developed through the literature to concisely guide future research. In the context of HMD VR, telepresence is defined as a participant being present within a parallel continuous streaming digital space as if the participant was actually a part of the digital space. We highlight the active role of VR sport gamers because of users' increasing level of autonomy in the VR environment due to the advancing VR technology in our definition. This actor-focused definition is grounded in Lee's (2004) presence theory, in which presence was defined as “a psychological state in which virtuality of experience is unnoticed” (p. 32). Our definition can help researchers further examine telepresence as a predictor of brand awareness in the VR gaming setting.

According to Cummings and Bailenson (2015), individuals may more likely perceive the virtual environment as a plausible space and themselves as located within it if spatial cues in the virtual environment have a logical consistency. Therefore, it is likely that individuals feel as if they are actually playing their sport in a stadium if the IGA is highly congruent with that sport during game play. For example, a Cleveland Cavaliers' fan who plays an NBA-themed VR game may feel as if he/she played basketball in Rocket Mortgage FieldHouse when he/she was surrounded by various Gatorade signage's in the virtual arena. Additionally, Bae et al. (2020) found that the characteristics of mixed reality, which represent presence, at cultural and artistic visitor attractions positively affect brand awareness. This suggests that gamers may recognize an IGA brand further when they feel as if they were in the gaming setting.

### Brand Awareness

Brand awareness has long been known as a measure of advertising effectiveness and predictor of future sales in a variety of settings (Aaker and Brown, 1972; Cornwell and Maignan, 1998). Brand awareness has been researched extensively in the sport sponsorship context and the main measures typically include consumer recall and recognition. Recall is typically unaided and researchers ask consumers to identify the advertisers from a website, event, or a video game without giving them further information. For instance, a researcher may ask a consumer who recently played a sport VR video game to identify all of the advertisers they saw during the course of playing the game in an open-ended format. Recognition typically requires consumers to identify official advertisers in a multiple-choice format from a list of actual advertisers and fake advertisers. For example, a researcher may ask study participants to identify all of the official advertisers after playing a sport video game from a list of both official advertisers and non-advertisers (Pham, 1992; Tripodi, 2001; Tripodi et al., 2003). While early advertising and sport sponsorship research has extensively investigated brand awareness as a measurement of advertising effectiveness, more recent research has focused on brand awareness as a measure of IGA effectiveness in sport video games (Cianfrone et al., 2008).

Initial IGA effectiveness studies utilizing brand awareness were first related to digital video games on the computer and gaming console platforms with more recent research focusing on mobile and VR games (Nelson, 2002; Cianfrone et al., 2008; Herrewijn and Poels, 2014; Wu et al., 2018). VR games and specifically sport VR games provide a highly interactive virtual advertising platform for advertisers to showcase their brands with IGA. As VR technology has improved, advertisers have been able to fully engage potential customers by increasing the interactivity of their embedded ads. For example, an advertiser in a basketball VR game may offer gamers the ability to change their branded shoes to a different model/color that has varying performance attributes. One type of basketball shoes may increase a player's speed while another type of shoes may increase a player's jumping ability and if the gamer is immersed in the virtual basketball world, they may physically feel these attributes during gameplay. This type of advertising experience is unique to VR games and advertising effectiveness research within VR and mobile gaming areas have shown a direct relationship between interactivity and brand awareness (Gao et al., 2009; Lee et al., 2014; Wu et al., 2018).

Both Cianfrone and Zhang (2013) and Kim et al. (2008), are seminal studies on the consumptive behaviors of sport video gamers and their relationship to consumer behavior of IGA. The researchers demonstrated that sport video game sponsorships/IGA were extremely effective in improving purchase intentions and this was especially the case for heavy gamers and sport consumers and those with a previous interest in the sponsoring brand. For instance, if a gamer playing a soccer video game like FIFA was already a loyal Apple consumer, Apple could potentially increase consumer purchase intentions with strategically placed IGA in the game. With that being said, while there is a good bit of research related to the effects of IGA on brand awareness in the traditional sport video game setting it appears that further investigation of how IGA influences brand awareness in the sport VR video game setting is warranted. Additionally, it may be important to examine VR gamer's attitudes toward the IGA embedded in games and previous brand loyalties. Similar to the issue of too much advertising at a live sports event, there is the potential for this negative effect with too much IGA in VR sport video games.

### Attitude Toward the IGA

Studies suggest that there is an increase in positive consumer attitude toward advertised brands when the IGA is congruent with the video game content (Lee and Faber, 2007; Chang et al., 2010). Since the IGA represents the brand being advertised, a positive attitude toward the IGA should translate to the same toward the brand, and vice versa. How a player feels about an IGA depends a lot on the nature of the in-game interaction and whether it is favorable or not. This too depends on whether the IGA enhances or impedes the gaming experience, the primary motivation for engaging with the VR medium, in this case.

Other factors that can affect the attitude toward VR IGA are perceived congruity of the IGA, perceived realism of the game play, and perceived intrusiveness of the IGA. Verberckmoes et al. (2016) showed that IGA congruity decreased perceived intrusiveness and increased realism, both of which contribute to a positive attitude toward the IGA. Contrary to this, other authors show that IGA that seem out of place with respect to other game environmental characteristics get noticed more, and hence are better recalled, suggesting benefits for incongruity (Verberckmoes et al., 2016). Advertisers want customers to notice the brand, like it, and then take action toward sustained engagement, ending in purchase for products. Incongruent IGA may upset the player, and since brand affinity is thus a goal, beyond awareness, perhaps as Lewis and Porter (2010) suggest a moderately congruent IGA may serve to both increase awareness and keep affinity positive.

## Discussion

### Intrusiveness and Attitude Toward the IGA

Two arguments may be presented following this comment: that experienced video game players, by being able to deliberately block out IGA, demonstrated that they were aware (at least momentarily) of the IGAs. One is only able to deliberately block out what they are aware of at that given moment in time. This should lead to great recall and recognition, but Lee and Faber (2007) argued the contrary. An alternative argument would be that the players are used to the game play and its environment containing the IGA (due to repeated practice). The participant players are thus able to block out IGAs, not because of an active awareness, but rather an active awareness of the game and a total lack of attention to anything not considered a part of the game. In either case, it is unlikely that the player would have a positive attitude toward this intrusive IGA in standard or VR game play.

Inversely, functional congruity occurs when the product category of the IGA is an essential element of game play (Gwinner and Eaton, 1999). This type of IGA may thus be tolerated and be even welcomed by participant players. The characteristic of interactivity of the IGA should be acceptable based on the same criteria as intrusiveness, only to the extent that it is functionally congruent. This should in turn lead to a harmonious game experience that may contribute to a positive participant experience, ultimately leading to an impactful attitude toward the IGA within an immersive VR sport gaming environment.

Where IGA intrusiveness and interactivity contribute to both a telepresence along with realism, and ultimately a more pleasurable game play experience, the attitude toward the IGA is expected to be positive. Functional congruity should help to bring this result because, while advertisers may be achieving brand promotion objectives through the IGA, they would simultaneously be contributing to the primary goal of gameplay. In short, with intrusiveness and attitude toward the IGA aligned the IGA would produce positive marketing outcomes, especially in sport VR experiences where advertisement inventory is maximized in real life.

### Interactivity Dimensions

Connectedness builds off of the Ha and James (1998) research and refers to the feeling of being linked to the product and company outside of the mobile or VR environments. Playfulness emphasizes entertainment and the inner joy experienced by the player of the mobile or VR game and is the first dimension of interactivity that emphasizes self-communication rather than interaction with others. The final dimension of interactivity is interpersonal communication, which refers to the degree to which the media platform allows users to communicate in a mediated, interactive environment. Additionally, researchers believe that improvement in technology such as VR headsets and improved graphics can significantly improve and enhance interpersonal communication. Regardless of the ongoing dissent related to the definition and measurement of interactivity in VR games, researchers agree that interactivity can lead to both a significant improvement in attitude toward an advertising brand and brand awareness of the IGA (Gao et al., 2009).

Being able to manipulate or change in-game objects is a key part of the interactivity of immersive VR sport experiences. An IGA can be embedded on an object with which the user interacts, as long as the IGA is thematically congruent with the object. An example would be a drink label (IGA) imprinted on an in-game drink bottle, such as Gatorade, as it will not be surprising that a drink bottle has a label. Interactivity in the sport game would require the user to hold, touch or change the object to achieve hydration.

Interactivity introduces dynamism that breaks the monotony of the normal, thus calling the user's attention. Following the argument that incongruent IGA's receive superior cognitive attention, it is therefore expected that the more interactive the IGA, the more incongruent, and hence the greater the likelihood that the user will interact with the object bearing the IGA, and finally, the greater the chance that the IGA will be remembered. In order to take advantage of the interactivity in this Gatorade example, the user would have to pick up the bottle. But what if the user, in the case of the bottle, does not want to drink? Offering them incentives during game play might lead them to pick up the bottle (interactive), what kind of incentive would they be offered if it does not contribute to the game play objective? Having players move aside embedded IGA objects could be regarded as interactive play that contributes to the game objective, to the extent that removing the barrier allows them to proceed within game play. Further IGA research is needed to understand how much cognitive involvement compares in this “negative-engagement” (remove obstacle) and a positive-engagement such as picking up to use within an immersive VR environment.

Additionally, De Pelsmacker et al. (2019) argues that interactivity of the IGA will distract from the game play. Li (2015) found domain experts who believe interactivity is neutral, and that what is important is thematic congruity. According to Li (2015), there would be no distractions when the quality of game design is good enough to achieve thematic congruity between IGA and the game. The limited-capacity model of attention (Lee and Faber, 2007) suggests that VR game players have limited cognitive resources, which they would prefer to deploy in the primary motive for playing the game, leisure. Besides subjective affective feelings, successful completion of VR in-game challenges may be confirmed by the attainment of high game scores. As Lee and Faber (2007) suggested, the more experienced players become, the more they learn to block out IGA, which they would regard as extraneous information toward the sport competition objective. In the end, IGA recall and recognition are likely to be low even in an immersive VR environment.

This research team recommends to differentiate between IGA that is designed to be interactive by the game designer, and whether or not it is engaged in by the user. This would be the case of those IGAs with dynamism of mobility, but without having anything to do with game play. From the advertiser's point of view, such IGAs would be more noticeable. However, since their interactivity is not a result of user-initiated actions, it may result in a lack of congruity, since it is an event outside normal game play or has a lack of congruity with the sport competition. Thus, advertiser generated IGAs might result in incongruity, and may lead to an eventual negative attitude toward the IGA.

### Implications and Future Research

This paper advances the VR sport marketing research discussion within the literature and provides a unique IGA congruity conceptual framework (see Figure 1) that can be tested in subsequent studies. Grounded in both the VR and sport advertising bodies of literature, this study attempts to address several emerging issues in the VR landscape. Additionally, as VR gaming becomes more affordable and accessible there will be significantly more opportunities for IGA within VR sport game development following the 2021 Oculus VR IGA beta testing. Consequently, the importance of studying the effectiveness of IGA in the VR gaming setting will become increasingly important. The *VR In-Game Advertising Congruity Framework* developed through a comprehensive review of literature will serve as a blueprint for researchers to further investigate VR IGA effectiveness in a variety of business settings. The theoretical framework created in this study builds off of the Pelsmacker et al. (2019) framework with inclusion of telepresence and brand awareness. The researchers also utilized seminal studies related to traditional sport video game consumer behavior to verify that this framework fits appropriately in a sport specific setting within a VR context.

A secondary, but very important theoretical implication of this study is a comprehensive review of the definition of telepresence, which is key to understanding the effects of IGA in VR games. While researchers have debated the specific definition of telepresence for decades now, the researchers in this study focused specifically on the interactivity aspect of telepresence within VR. In the context of IGA within VR gaming, this involves a feeling that the participant is actually in the digital space and the influence of telepresence on brand awareness warrants further investigation (Faiola et al., 2013; Herrewijn and Poels, 2014). Again, in the context of HMD VR, this research team defines telepresence through the literature as a participant being present within a parallel continuous streaming digital space as if the participant was actually a part of the digital space. As the VR sport gaming experience continues to improve, researchers will have additional opportunities to examine telepresence and how we should look at IGA within the VR gaming environment space under this cleaned definition.

In addition to the effect of telepresence on brand awareness, the conceptual framework proposed in this study (see Figure 1) also further examines the effect of the IGA's congruity on brand awareness. Lee and Faber (2007) suggested four dimensions of congruity when considering the relationship between the product category of the embedded IGA and the content of the game, within which it is embedded: (1) Functional congruity is when the advertised product category is used in the game. (2) Life congruity is when both the product category and the game content are designed for the sociodemographic group, while (3) image congruity is when the image of both the product category of the IGA match the same of the game focus. Finally, (4) advertising congruity is when the IGA product category is appropriate for the game context.

Some authors are of the opinion that congruency between in-game elements produces a superior impact on memory (Lambert, 1980; Shamdasani et al., 2001; Moorman et al., 2007; Rodgers, 2013), especially when people have a pre-existing mental schema against which they compare new information and experiences. A match between the new information and existing schema, where the former is absorbed by the latter results in a great recall of the congruent information. The aforementioned authors found that incongruent information has a comparatively inferior effect on memory to congruent information, which suggest negative brand awareness within sport VR games.

Others argue that incongruent information has a superior effect on the memory, because the novelty and the uniqueness of the out-of-place information make them noticeable (Heckler and Childers, 1992; Forehand et al., 2002; Russell, 2002; Dimofte et al., 2011). It is in the attempt to make sense of the incongruous that the cognition becomes more engaged, and thus make it easy to recall (Srull and Wyer, 1979; Mandler and Shebo, 1982). Lee and Faber (2007) believed that neither of the two above (congruency or incongruency *per se*) is an adequate explanation for the recall ability of the memory. On the one hand, they argued, it depends on the degree of either congruity or incongruity. Higher levels are remembered more easily than low or moderate levels. On the other hand, recall, they suggested, depends on how much attentional or processing resources are left over after being dedicated to the primary task e.g., an online game. Thus, in order for memory resources to be allocated to the new information, its incongruency ought to be particularly large in order for the brain to undertake the cognitive elaboration to make sense of it. The more incongruent the brand, the greater will be the brand recall and recognition (memory). This may contradict some traditional sport sponsorship/brand awareness research where sponsor/brand fit is very important (Kim et al., 2008; Cianfrone and Zhang, 2013) and further research is needed to test the *VR In-Game Advertising Congruity Framework* in order to determine the optimum line that IGA incongruity can be pushed without resulting in a negative attitude toward the IGA within an immersive VR environment.

The level of involvement of the player within the game can also affect the impact of congruity on memory. Players who are more involved, which in VR can be operationalized by the interactivity of either the game or the IGA, will be so focused on the game that they tend to block out any information considered extraneous, in this case, an incongruous IGA. Thus, it is hypothesized for future research that more experienced VR gamers should exhibit lower recall than moderate or less involved VR gamers. For example, experienced VR gamers playing FIFA may have high fan identification for their selected club and be able to block out more incongruous IGA based on familiarity with the competition and immersive VR setting.

Considering the different ways in which congruity has been conceptualized will be helpful in understanding the interactions within VR, with the special characteristics of this immersive media. Lee and Faber (2007) identified some of these conceptualizations, which either relate to the IGA itself, or refer to aspects of the IGA as well as the game context. They include the relationships between the visual and the verbal elements of the IGA; the sponsor and the advertised event; the IGA and the context where it appears; the relationship between the modality (the audio and the visual placements of the IGA) and the plot.

Future research needs to empirically examine each of the variable relationships within the *VR In-Game Advertising Congruity Framework* under the VR sport gaming context. Given the multidisciplinary nature of the research framework, its examination should not be restricted to just a sport marketing context, and rather multidisciplinary VR efforts will be optimal. Although the VR gaming industry has been exponentially growing in the last few years, there is room for improvement for both hardware and software within the $7.7 billion USD industry (Wood, 2020) that is expected to reach $57.55 billion USD by 2027 (Fortune Business Insights, 2021). Multidisciplinary contribution can enhance the collective level of understanding that researchers have about human behaviors in an extensive range of contexts (Kim et al., 2008). For example, collaborations among scholars in psychology, marketing, industrial engineering, and computer software science can produce rich findings and provide a great deal of insights for both researchers and practitioners.

This *VR In-Game Advertising Congruity Framework* can be tested using various VR games, and through group difference testing such as ANOVA, in order to examine framework variable similarities across different games genres (e.g., sport, action, action-adventure, military, role-playing games, etc.). Researchers can also utilize eye tracking built into VR HMDs such as the HTC VIVE Pro Eye with Tobii analytical software on either commercially available games or games in development creating further research opportunities. For example, researchers can develop a VR basketball game that allows IGA to be embedded or excluded in a way to manipulate the level of intrusiveness. VR players can be exposed to either a highly intrusive condition or unintrusive condition while playing the game. Following the completion of the game, the participants can complete a *VR In-Game Advertising Congruity Framework* guided survey or interview on their gaming experience, which includes IGA brand awareness and attitude toward the IGA. Additionally, researchers can explore framework ANOVA relationships to eye tracking analytics measuring gamer glances on the IGA through heat maps.

Finally, from a practitioner perspective, this study aims to benefit video game developers and advertisers. The proposed *In-Game Advertising Congruity Framework* of this study and comprehensive literature review provide a basis for video game developers and advertisers to make informed decisions regarding IGA in VR video games. This work is unique as there is a paucity of research within the literature on sport video games and none to date within the VR sport game context. The *In-Game Advertising Congruity Framework* provides practitioners and academics alike with a baseline to measure fandom with respect to irrational choice of sport consumption and if purchase behaviors extend to a VR sport gaming experience. While it is difficult to conceptualize the role of each framework variable without empirical evidence, the context and role of IGA in VR reveals a unique boundary condition of existing theoretical frameworks. Additionally, VR gamers will potentially benefit from this study through more effective IGAs that are less intrusive and offer gamers targeted advertisements related to products that they may be interested in purchasing.

## Author Contributions

All authors listed have made a substantial, direct and intellectual contribution to the work, and approved it for publication.

## Funding

The publication fees for this article were funded through the Feliciano School of Business at Montclair State University.

## Conflict of Interest

The authors declare that the research was conducted in the absence of any commercial or financial relationships that could be construed as a potential conflict of interest.

## Publisher's Note

All claims expressed in this article are solely those of the authors and do not necessarily represent those of their affiliated organizations, or those of the publisher, the editors and the reviewers. Any product that may be evaluated in this article, or claim that may be made by its manufacturer, is not guaranteed or endorsed by the publisher.

## References

[B1] Aaker D. A. Brown P. K. (1972). Evaluating vehicle source effects. J. Advert. Res. 12, 11–16.

[B2] Bae S. Jung T. H. Moorhouse N. Suh M. Kwon O. (2020). The influence of mixed reality on satisfaction and brand loyalty in cultural heritage attractions: a brand equity perspective. Sustainability 12:2956. 10.3390/su12072956

[B3] Biocca F. Levy M. R. (1995). Communication in the Age of Virtual Reality. Lawrence Erlbaum Associates.

[B4] Chang Y. Yan J. Zhang J. Luo J. (2010). Online in-game advertising effect: examining the influence of a match between games and advertising. J. Interact. Advert. 11, 63–73. 10.1080/15252019.2010.10722178

[B5] Cianfrone B. Trail G. Zhang J. Lutz R. (2008). Effectiveness of in game advertisements in sport video games: an experimental inquiry on current gamers. Int. J. Sport Commun. 1, 195–218. 10.1123/ijsc.1.2.195

[B6] Cianfrone B. A. Zhang J. J. (2013). The impact of gamer motives, consumption, and in-game advertising effectiveness: a case study of football sport video games. Int. J. Sport Commun. 6, 325–347. 10.1123/ijsc.6.3.325

[B7] Cornwell T. B. Maignan I. (1998). An international review of sponsorship research. J. Advert. 27, 1–44. 10.1080/00913367.1998.10673539

[B8] Cummings J. J. Bailenson J. N. (2015). How immersive is enough? A meta-analysis of the effect of immersive technology on user presence. Media Psychol. 19, 272–309. 10.1080/15213269.2015.1015740

[B9] De Pelsmacker P. Dens N. Verberckmoes S. (2019). How ad congruity and interactivity affect fantasy game players' attitude toward in-game advertising. J. Electron. Commer. Res. 20, 55–74.

[B10] Dimofte C. Goodstein R. Kalra A. (2011). “Increasing attention at what cost? Consumer reactions to context-sensitive advertising,” in NA—Advances in Consumer Research, Vol. 39, eds R. Ahluwalia, T. L. Chartrand, and R. K. Ratner (Duluth, MN: Association for Consumer Research), 457.

[B11] Faiola A. Newlon C. Pfaff M. Smyslova O. (2013). Correlating the effects of flow and telepresence in virtual worlds : Enhancing our understanding of user behavior in game-based learning. Comput. Hum. Behav. 29, 1113–1121. 10.1016/j.chb.2012.10.003

[B12] Forehand M. R. Deshpandé R. Reed A. II. (2002). Identity salience and the influence of differential activation of the social self-schema on advertising response. J. Appl. Psychol. 87, 1086–1099. 10.1037/0021-9010.87.6.1086 12558216

[B13] Fortune Business Insights (2021). Virtual Reality Market to Reach USD 57.55 Billion by 2027: Surging Adoption of VR Technology by Companies and Various Sectors to Propel Market Growth. Available online at: https://www.globenewswire.com/en/news-release/2021/05/12/2228178/0/en/Virtual-Reality-Market-to-Reach-USD-57-55-Billion-by-2027-Surging-Adoption-of-VR-Technology-by-Companies-and-Various-Sectors-to-Propel-Market-Growth-Fortune-Business-Insights.html

[B14] Gao Q. Rau P. L. P. Salvendy G. (2009). Perception of interactivity: affects of four key variables in mobile advertising. Int. J. Hum. Comput. Interact. 25, 479–505. 10.1080/10447310902963936

[B15] Gwinner K. P. Eaton J. (1999). Building brand image through event sponsorship: the role of image transfer. J. Advert. 28, 47–57. 10.1080/00913367.1999.10673595

[B16] Ha L. (1996). Advertising clutter in consumer magazines: dimensions and effects. J. Advert. Res. 36, 76–85.

[B17] Ha L. James E. (1998). “Interactivity reexamined: an analysis of business websites,” in Proceedings of the Conference of the American Academy of Advertising (Pullman, WA).

[B18] Hall A. (2003). Reading realism: audiences' evaluations of the reality of media texts. J. Commun. 53, 624–641. 10.1111/j.1460-2466.2003.tb02914.x

[B19] Heckler S. E. Childers T. L. (1992). The role of expectancy and relevancy in memory for verbal and visual information: what is incongruency? J. Consum. Res. 18, 475–492. 10.1086/209275

[B20] Held R. M. Durlach N. I. (1992). Telepresence. Presence Teleoperators Virtual Environ. 1, 109–112. 10.1121/1.404500

[B21] Herrewijn L. Poels K. (2014). “Rated a for advertising: a critical reflection on in-game advertising,” in Handbook of Digital Games, eds M. C. Angelides and H. Agius (Hoboken, NJ: John Wiley and Sons), 305–335. 10.1002/9781118796443.ch11

[B22] Hong S. Magnusen M. (2017). From virtual reality to reality: examining the relationship between sport video gaming and sport consumption behaviors. J. Phys. Sport Manag. 8, 41–49. 10.5897/JPESM2016.0272

[B23] Hvass J. Larsen O. Vendelbo K. Nilsson N. Nordahl R. Serafin S. (2017). “Visual realism and presence in a virtual reality game,” in Proceedings of the 3DTV Conference: The True Vision-Capture, Transmission and Display of 3D Video (3DTV-CON) (Copenhagen: IEEE Xplore), 1–4. 10.1109/3DTV.2017.8280421

[B24] Hyun M. Y. O'Keefe R. M. (2012). Virtual destination image: testing a telepresence model. J. Bus. Res. 65, 29–35. 10.1016/j.jbusres.2011.07.011

[B25] Kahneman D. (1973). Attention and Effort, Vol. 1063, Hoboken, NJ: Prentice-Hall.

[B26] Karrh J. A. McKee K. B. Pardun C. J. (2003). Practitioners' evolving views on product placement effectiveness. J. Advert. Res. 43, 138–149. 10.2501/JAR-43-2-138-149

[B27] Kim D. Ko Y. J. (2019). The impact of virtual reality (VR) technology on sport spectators' flow experience and satisfaction. Comput. Hum. Behav. 93, 346–356. 10.1016/j.chb.2018.12.040

[B28] Kim T. Biocca F. (1997). Telepresence via television: two dimensions of telepresence may have different connections to memory and persuasion. J Comput Mediat Commun. 3. 10.1111/j.1083-6101.1997.tb00073.x

[B29] Kim Y. Walsh P. Ross S. D. (2008). An examination of the psychological and consumptive behaviors of sport video gamers. Sport Mark. Q. 17, 44–53.

[B30] Klein L. R. (2003). Creating virtual product experiences: the role of telepresence. J. Interact. Mark. 17, 41–55. 10.1002/dir.10046

[B31] Krcmar M. Farrar K. McGloin R. (2011). The effects of video game realism on attention, retention and aggressive outcomes. Comput. Hum. Behav. 27, 432–439. 10.1016/j.chb.2010.09.005

[B32] Lambert D. (1980). Transactional analysis as a congruity paradigm for advertising recall. J. Advert. 9, 37–45. 10.1080/00913367.1980.10673318

[B33] Lee J. Park H. Wise K. (2014). Brand interactivity and its effects on the outcomes of advergame play. New Media Soc. 16, 1268–1286. 10.1177/1461444813504267

[B34] Lee K. M. (2004). Presence, explicated. Commun. Theor. 14, 27–50. 10.1111/j.1468-2885.2004.tb00302.x

[B35] Lee M. Faber R. J. (2007). Effects of product placement in on-line games on brand memory. J. Advert. 36, 75–90. 10.2753/JOA0091-3367360406

[B36] Lewis B. Porter L. (2010). In-Game advertising effects: examining player perceptions of advertising schema congruity in a Massively Multiplayer Online Role-Playing Game. J. Interact. Advert. 10, 46–60. 10.1080/15252019.2010.10722169

[B37] Li H. Edwards S. M. Lee J. H. (2002). Measuring the intrusiveness of advertisements: scale development and validation. J. Advert. 31, 37–47. 10.1080/00913367.2002.10673665

[B38] Li X. (2015). Playful advertising: In-game advertising for virtual reality games (Master's thesis). KTH Royal Institute of Technology, Stockholm, Sweden.

[B39] Liu Y. Shrum L. (2002). What is interactivity and is it always such a good thing? Implications of definition, person, and situation for the influence of interactivity on advertising effectiveness. J. Advert. 31, 53–64. 10.1080/00913367.2002.10673685

[B40] Lombard M. Ditton T. (1997). At the heart of it all: the concept of presence. J. Comput. Mediat. Commun. 3, 1–15. 10.1111/j.1083-6101.1997.tb00072.x

[B41] Malliet S. (2006). An exploration of adolescents' perceptions of videogame realism. Learn. Media Technol. 31, 377–394. 10.1080/17439880601021983

[B42] Mandler G. Shebo B. J. (1982). Subitizing: an analysis of its component processes. J. Exp. Psychol. Gen. 111, 1–22. 10.1037/0096-3445.111.1.1 6460833

[B43] McGloin R. Farrar K. Krcmar M. (2013). Video games, immersion, and cognitive aggression: does the controller matter? Media Psychol. 16, 65–87. 10.1080/15213269.2012.752428

[B44] Mcgloin R. Farrar K. M. Fishlock J. (2015). Triple whammy! Violent games and violent controllers: investigating the use of realistic gun controllers on perceptions of realism, immersion, and outcome aggression. J. Commun. 65, 280–299. 10.1111/jcom.12148

[B45] McGloin R. Farrar K. M. Krcmar M. (2011). The impact of controller naturalness on spatial presence, gamer enjoyment, and perceived realism in a tennis simulation video game. Presence Teleoper. Virtual Environ. 20, 309–324. 10.1162/PRES_a_00053

[B46] Mollen A. Wilson H. (2010). Engagement, telepresence and interactivity in online consumer experience: reconciling scholastic and managerial perspectives. J. Bus. Res., 63, 919–925. 10.1016/j.jbusres.2009.05.014

[B47] Moorman M. Neijens P. C. Smit E. G. (2007). The effects of program involvement on commercial exposure and recall in a naturalistic setting. J. Advert. 36, 121–137. 10.2753/JOA0091-3367360109

[B48] Murphy R. (2021). How Much Money Does EA Sports Make From FIFA and Ultimate Team? Available online at: https://www.goal.com/en-ae/news/how-much-money-does-ea-sports-make-from-fifa-ultimate-team/r1tbutqcbjhx19gkz54rtrp68 (accessed August 31, 2021).

[B49] Nah F. F.-H. Eschenbrenner B. DeWester D. (2011). Enhancing brand equity through flow and telepresence. MIS Q. 35, 731–A19. 10.2307/23042806

[B50] Nelson M. (2002). Recall of brand placements in computer/video games. J. Advert. Res. 42, 80–92. 10.2501/JAR-42-2-80-92

[B51] Nelson M. R. Keum H. Yaros R. A. (2004). Advertainment or adcreep? Game players' attitudes toward advertising and product placements in computer games. J. Interact. Advert. 4, 1–30. 10.1080/15252019.2004.10722090

[B52] Nelson M. R. Yaros R. A. Keum H. (2013). Examining the influence of telepresence on spectator and player processing of real and fictitious brands in a computer game. J. Advert. 35, 87–99. 10.2753/JOA0091-3367350406

[B53] Olshavsky R. W. (1994). “Attention as an epiphenomenon: some implications for advertising,” in Attention, Attitude and Affect in Response to Advertising, eds E. M. Clark, T. C. Brock, and D. W. Stewart (Psychology Press), 97–106.

[B54] Olson M. (2021). Facebook Tiptoes Into Advertising Inside VR Games. The Information. Available online at: https://www.theinformation.com/articles/facebook-tiptoes-into-advertising-inside-vr-games (accessed August 31, 2021).

[B55] Papadopoulos S. (2020). Effects of in-game advertising on brand awareness in virtual reality game interactions (Master's Thesis). KTH Royal Institute of Technology. Available online at: https://www.diva-portal.org/smash/get/diva2:1466951/FULLTEXT01.pdf

[B56] Pelsmacker P. D. Dens N. Verberckmoes S. (2019). How ad congruity and interactivity affect fantasy game players' attitude toward in-game advertising. J. Electron. Commer. Res. 20, 55–74.

[B57] Pham T. M. (1992). Effects of involvement, arousal, and pleasure on the recognition of sponsorship stimuli. Adv. Consum. Res. 19, 85–93.

[B58] Poels K. Janssens W. Herrewijn L. (2013). Play buddies or space invaders? Players' attitudes toward in-game advertising. J. Advert. 42, 204–218. 10.1080/00913367.2013.774600

[B59] Potter W. J. (1988). Perceived reality in television effects research. J. Broadcast. Electron. Media 32, 23–41. 10.1080/08838158809386682

[B60] Riedel A. S. Weeks C. S. Beatson A. T. (2018). Am I intruding? Developing a conceptualisation of advertising intrusiveness. J. Mark. Manage. 34, 750–774. 10.1080/0267257X.2018.1496130

[B61] Rodgers S. (2013). The effects of sponsor relevance on consumer reactions to Internet sponsorships. J. Advert. 32, 67–76. 10.1080/00913367.2003.10639141

[B62] Russell C. A. (2002). Investigating the effectiveness of product placements in television shows: the role of modality and plot connection congruence on brand memory and attitude. J. Consum. Res. 29, 306–318. 10.1086/344432

[B63] Shamdasani P. N. Stanaland A. J. S. Tan J. (2001). Location, location, location: insights for advertising placement on the web. J. Advert. Res. 41, 7–21. 10.2501/JAR-41-4-7-21

[B64] Slater M. Usoh M. (1993). Representations systems, perceptual position, and presence in immersive virtual environments. Presence Teleoperators Virtual Environ. 2, 221–233. 10.1162/pres.1993.2.3.221

[B65] Srull T. K. Wyer R. (1979). The role of category accessibility in the interpretation of information about persons: some determinants and implications. J. Pers. Soc. Psychol. 37, 1660–1672. 10.1037/0022-3514.37.10.1660

[B66] Steuer J. (1992). Defining virtual reality: dimensions determining telepresence. J. Commun. 42, 73–93. 10.1111/j.1460-2466.1992.tb00812.x

[B67] Steuer J. (1995). “Defining virtual reality: dimensions determining telepresence,” in Communication in the Age of Virtual Reality, eds F. Biocca and M. Levy (Lawrence Erlbaum and Associates), 33–56.

[B68] Tamborini R. (2000). “The experience of telepresence in violent video games,” in Proceedings of the 86th Annual Convention of the National Communication Association (Seattle, WA).

[B69] Tavinor G. (2019). “Towards an analysis of virtual realism,” in Proceedings of the Digital Games Research Association (DiGRA) Conference (Kyoto).

[B70] Tripodi J. (2001). Sponsorship-a confirmed weapon in the promotional armory. Int. J. Sports Mark. Spons. 3, 95–116. 10.1108/IJSMS-03-01-2001-B007

[B71] Tripodi J. A. Hirons M. Bednall D. Sutherland M. (2003). Cognitive evaluation: prompts used to measure sponsorship awareness. Int. J. Res. Mark. 45, 435–455. 10.1177/147078530304500401

[B72] Verberckmoes S. Poels K. Dens N. Herrewijn L. De Pelsmacker P. (2016). When and why is perceived congruity important for in-game advertising in fantasy games? Comput. Hum. Behav. 64, 871–880. 10.1016/j.chb.2016.07.062

[B73] Wood L. (2020). Global Virtual Reality in Gaming Market (2020 to 2025)—Growth, Trends, and Forecast. Research and Markets. Available online at: https://www.globenewswire.com/news-release/2020/07/22/2065574/0/en/Global-Virtual-Reality-In-Gaming-Market-2020-to-2025-Growth-Trends-and-Forecast.html

[B74] Wu C. Tsung-kuang E. M. Tien T. W. (2018). The effect of in-game brand placement prominence and players' flow experience on brand recall: the moderating role of game genre. Int. J. Bus. Soc. Sci. Res. 9, 70−75.

[B75] Wu J. Li P. Rao S. (2008). Why they enjoy virtual game worlds? An empirical investigation. J. Electron. Commer. Res. 9, 219−230.

[B76] Wu J. Liu D. (2007). The effects of trust and enjoyment on intention to play online games. J. Electron. Commer. Res. 8, 128−140.

